# Glycolysis in Chronic Liver Diseases: Mechanistic Insights and Therapeutic Opportunities

**DOI:** 10.3390/cells12151930

**Published:** 2023-07-26

**Authors:** Hengdong Qu, Junli Liu, Di Zhang, Ruoyan Xie, Lijuan Wang, Jian Hong

**Affiliations:** Department of Pathophysiology, School of Medicine, Jinan University, Guangzhou 510632, China; quhengdong@stu2021.jnu.edu.cn (H.Q.);

**Keywords:** chronic liver disease, glycolysis, HIF-1α, metabolic reprogramming, immune activation, PKM2, therapeutic target

## Abstract

Chronic liver diseases (CLDs) cover a spectrum of liver diseases, ranging from nonalcoholic fatty liver disease to liver cancer, representing a growing epidemic worldwide with high unmet medical needs. Glycolysis is a conservative and rigorous process that converts glucose into pyruvate and sustains cells with the energy and intermediate products required for diverse biological activities. However, abnormalities in glycolytic flux during CLD development accelerate the disease progression. Aerobic glycolysis is a hallmark of liver cancer and is responsible for a broad range of oncogenic functions including proliferation, invasion, metastasis, angiogenesis, immune escape, and drug resistance. Recently, the non-neoplastic role of aerobic glycolysis in immune activation and inflammatory disorders, especially CLD, has attracted increasing attention. Several key mediators of aerobic glycolysis, including HIF-1α and pyruvate kinase M2 (PKM2), are upregulated during steatohepatitis and liver fibrosis. The pharmacological inhibition or ablation of PKM2 effectively attenuates hepatic inflammation and CLD progression. In this review, we particularly focused on the glycolytic and non-glycolytic roles of PKM2 in the progression of CLD, highlighting the translational potential of a glycolysis-centric therapeutic approach in combating CLD.

## 1. Introduction

Chronic liver disease (CLD) encompasses a broad spectrum of liver diseases, ranging from viral hepatitis, alcoholic liver disease (ALD), and nonalcoholic fatty liver disease (NAFLD) to end-stage hepatic conditions including nonalcoholic steatohepatitis (NASH), liver fibrosis, hepatocellular carcinoma (HCC), and cholangiocarcinoma (CCA) [1]. Due to the growing prevalence of metabolic syndromes, including obesity and hyperlipidemia, NAFLD has become the most prevalent CLD, affecting more than 25% of the adult population worldwide [2]. Currently, there are no approved therapeutics for certain CLDs, such as NASH and liver fibrosis, which is partly limited by the lack of understanding of their complicated pathogenesis and druggable targets [3,4].

Liver is the most vital organ for glucose homeostasis, where it generates glucose during fasting and reserves glucose postprandially [5,6]. During the progression of CLD, hepatocyte injury and chronic low-grade inflammation lead to metabolic dysfunction, which causes an excessive accumulation of lipids and aberrant activation of metabolic pathways in the liver, including enhanced aerobic glycolysis [7,8]. On one hand, aerobic glycolysis promotes the pro-inflammatory activation of immune cells, which perpetuates hepatic inflammation and liver injury [9]. On the other hand, HCC encompasses enhanced aerobic glycolysis to support the proliferation, metastasis, and drug resistance of HCC cells [10]. Growing evidence has highlighted the significance of aerobic glycolysis in the progression of CLDs, suggesting that targeting abnormal glycolytic flux may serve as an effective strategy to combat CLDs [11,12,13,14].

**Figure 1:**
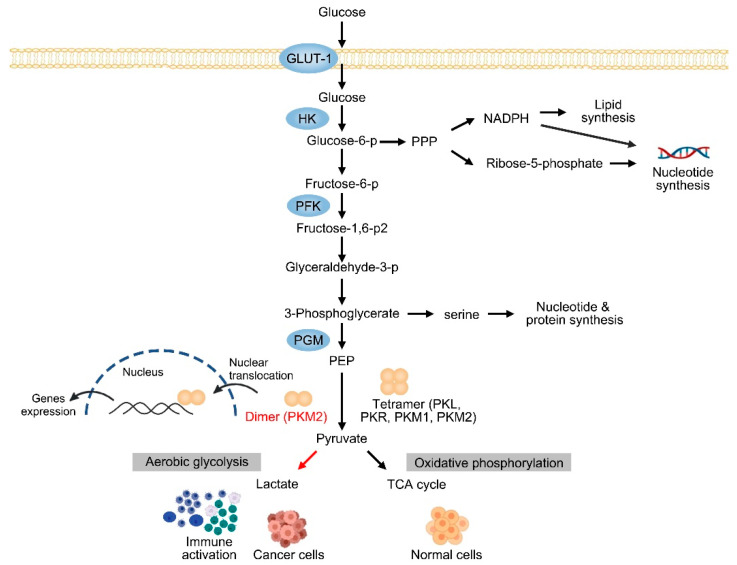
Pyruvate kinase isoforms in metabolic reprogramming.

Several glycolytic mediators have been reported to regulate the progression of CLDs. Pyruvate kinase (PK) is a rate-limiting enzyme that catalyzes the final step of glycolysis. It converts phosphoenolpyruvate (PEP) into pyruvate and supports ATP production during glycolysis. Unlike mitochondrial respiration, PK produces ATP independent of oxygen supply, which allows organs to survive under hypoxic conditions [15]. Due to the unique metabolic requirements of tissues, the expression levels of each pyruvate kinase isozyme vary substantially in both kinetics and regulatory mechanisms. There are four subtypes of PKs, ranging from PKL and PKR encoded by *PKLR* gene to PKM1 and pyruvate kinase M2 (PKM2) encoded by the *PKM* gene [16]. Unlike other isoforms that exclusively function as hyperactive tetramers and promote metabolic flux toward oxidative phosphorylation (OXPHOS), PKM2 contains a less active monomeric and dimeric form, which shifts the metabolite from OXPHOS to aerobic glycolysis [17]. In addition, the PKM2 dimer can translocate to the nucleus and act as a transcriptional coactivator to regulate gene expression [18]. Interestingly, PKM2 directly binds to HIF-1α and promotes the transcription of a series of glycolytic genes, including PKM2, thereby forming a positive feedback loop for aerobic glycolysis (Figure 1) [19].

Owing to its unique properties, PKM2 is preferentially upregulated in immune and cancerous cells, which display high levels of nucleic acid synthesis [20]. Previous works on PKM2 have mainly investigated its effect on the metabolism, proliferation, and migration of tumor cells [21]. Recent studies have shown that PKM2 is involved in immune activation via reprogramming glycolysis [22,23]. In this review, we summarize the neoplastic and non-neoplastic role of aerobic glycolysis in the progression of CLD by particularly focusing on PKM2, highlighting the translational potential of applying PKM2 as a druggable target to combat CLDs.

## 2. The Expression of PKM2 in CLDs

In line with the dynamic metabolic conditions and characteristics of different tissues, levels of PK isoforms are highly regulatory and tissue-specific. PKL and PKR are primarily expressed in the healthy liver, intestine, and red blood cells. PKM1 is expressed in adult tissues, including the bone and brain, whereas PKM2 is expressed in embryonic cells, undifferentiated tissues, and tumors [21]. Consistent with this notion, PKM2 expression is hardly detectable in healthy livers but is dramatically upregulated in liver cancer [24,25]. Interestingly, although a switch from PKM1 to PKM2 regulated by the alternative splicing of PKM was observed in many types of tumor, PKM2 was the prominent isoform of PKM both in normal liver and HCC [26]. During DEN/CCl_4_- or STZ/HFD-induced murine hepatocarcinogenesis, the level of PKM2 in the plasma ectosome gradually increased before tumor formation. Moreover, PKM2 was significantly enriched in ectosomes from patients with HCC compared to healthy donors, indicating that PKM2 may serve as an early diagnostic marker for HCC [27]. In patients with HCC, PKM2 is highly upregulated in tumor tissues and is positively correlated with poor prognosis [28,29,30]. Furthermore, the overexpression of PKM2 in HCC is correlated with a high TNM stage and level of vascular invasion, and patients who are positive for PKM2 expression have an increased incidence of postsurgical HCC recurrence [31,32].

Liver cirrhosis, the progressive stage of liver fibrosis, is recognized as a key mediator in the pathogenesis of liver cancer as it progresses to HCC in up to 90% of patients [33]. Interestingly, PKM2 expression is significantly increased in precancerous cirrhotic livers and strongly associated with an elevated risk of developing HCC [34]. Moreover, the level of hepatic PKM2 is higher in cirrhotic HCC than in non-cirrhotic HCC, suggesting that PKM2 plays an important role in the regulation of the precancerous and tumor microenvironments of HCC [35]. The expansion of PD-L1^+^ tumor-associated macrophages is a critical driver of the immune escape of HCC and correlates with poor prognosis in HCC patients [36]. Notably, PKM2 is overexpressed in PD-L1^+^ glycolytic macrophages, and PD-L1 blockade liberates the intrinsic antitumorigenic properties of PKM2^+^ macrophages, indicating that PKM2 may serve as an indicator for precise anticancer therapy [37]. PKM2 is also upregulated in CCA tissues compared to healthy liver tissues and is positively correlated with the poor prognosis of CCA patients [38]. Serum PKM2 levels are elevated in patients with precancerous cholangitis, and CCA and can be used to discriminate between benign and malignant stages of disease with high specificity and sensitivity [39]. These studies suggest that PKM2 is a key player in the progression of liver cancer and may serve as an effective prognostic and predictive biomarker.

In addition to cancerous conditions, PKM2-mediated aerobic glycolysis plays a critical role in inflammatory disorders and nonneoplastic liver diseases [19,23]. The expression of PKM2 is upregulated in HSC in the context of liver fibrosis and is overexpressed in hepatic macrophages and Th17 cells during NAFLD/NASH development [40,41,42]. Of note, serum and hepatic levels of PKM2 are increased in both metabolic syndrome and NAFLD, but they decreased after Roux-en-Y gastric bypass surgery, one of the most commonly performed weight-loss procedures, implying that systematic PKM2 expression may represent the disease stage of NAFLD [43]. Currently, there are no effective noninvasive diagnostic methods for NAFLD, NASH, or liver fibrosis. The pattern of PKM2 expression in inflammatory liver diseases may lead to the development of novel biomarkers for accurate diagnosis, either independently or along with liver biopsy, which is the gold standard for NASH diagnosis [44,45]. Collectively, it would be of great clinical significance to characterize the expression of PKM2 in CLDs for the development of novel diagnostic and prognostic biomarker.

## 3. Current Status and Challenge of Liver Cancer

The liver is the sixth most prevalent site of primary cancers, including HCC and CCA. Liver cancer is the fourth leading cause of cancer-related deaths worldwide owing to its high incidence of postsurgical recurrence and metastasis [46]. In particular, HCC accounts for 85–90% of liver cancers [47]. Owing to the increased global incidence of metabolic syndrome, NAFLD has become the most prominent cause and risk factor for HCC in numerous developed countries [48]. Traditionally, systemic therapies, including sorafenib or lenvatinib, have been practiced as the first-line therapy. Recently, ICIs have revolutionized HCC treatment, with a significant increase in patient survival [49]. A combination of Atezolizumab with Bevacizumab was approved as first-line HCC therapy in 2020. Tremelimumab and durvalumab were also approved as first-line HCC therapies in 2022. Despite these major advances, NAFLD has been reported to limit the therapeutic efficacy of ICI in treating HCC, and a better stratification system based on different individuals is still needed to guide clinical decision making [50].

### 3.1. PKM2 in HCC

Aerobic glycolysis is one of the most prominent features of liver cancer which supports a broad range of oncogenic regulation, including proliferation, metastasis, immunosuppression, and drug resistance [7]. In this section, we introduce recent advances in PKM2-directed glycolysis for HCC formation and progression. Several mechanisms have been reported to modulate PKM2-mediated aerobic glycolysis and the progression of HCC. HSP90 promotes the Warburg effect and proliferation of HCC cells via direct binding to PKM2 and phosphorylates it at Thr-328, which is a site that is critical for sustaining PKM2 stability [51]. Circular RNA MAT2B sponges miR-338-3p and promotes the expression of PKM2, thereby enhancing aerobic glycolysis and HCC progression under hypoxia [52]. Yu et al. found that MTR4, an RNA helicase, drives cancer metabolism and HCC progression by ensuring the alternative splicing of specific glycolytic genes, including PKM2 [53]. HIF-1α plays a critical role in regulating the transcription of glycolytic genes, especially in tumor microenvironments, including HCC [54,55]. Under hypoxia, YAP binds to HIF-1α in the nucleus, which thereby maintains HIF-1α stability and the aerobic glycolysis of HCC. Moreover, HIF-1α binds to *PKM* mRNA and directly activates the transcription of PKM2, accelerating the glycolysis of HCC cells [56]. Meanwhile, PKM2 is known to regulate HIF-1α transactivation, which results in an upregulation of several glycolytic genes, including LDHA and PKM2 [57,58]. This positive feedback loop may further fuel aerobic glycolysis and cause drug resistance to PKM2-targeting therapy [59].

The nuclear translocation of PKM2 is considered an indispensable course in the stimulation of aerobic glycolysis, progression, and drug resistance in HCC. Enhanced aerobic glycolysis is associated with HCC resistance to sorafenib, whereas the disruption of PKM2-associated glycolysis increases apoptosis and re-sensitizes resistant tumor cells to sorafenib [60]. A study showed that PRMT6 promotes PKM2 nuclear translocation, leading to increased aerobic glycolysis in HCC, while the addition of 2-DG (a well-known inhibitor of glycolysis) sufficiently reverses PRMT6 deficiency-mediated tumor progression and sorafenib resistance [61]. Zhou et al. reported that GTPBP4 induces PKM2 sumoylation and dimer formation. Dimeric PKM2 further translocates into the nucleus, thereby facilitating EMT and aerobic glycolysis in HCC via the STAT3 signaling pathway [62]. Myofibroblasts MyD88-mediated CCL20 secretion promoted PKM2 nuclear translocation and aerobic glycolysis in HCC cells via an ERK-dependent signaling pathway [63]. Additionally, PKM2 has been reported to fuel HCC metastasis and inhibit autophagy through the JAK/STAT3 pathway [64].

PKM2 also contributes to the development of an immunosuppressive microenvironment during HCC progression [65]. PKM2 levels were positively correlated with the levels of immune inhibitory cytokine and immune cell infiltration in HCC [28]. Lu et al. reported that PD-L1^+^ macrophages display high levels of glycolysis via the PKM2/HIF-1α axis triggered by fibronectin 1 derived from HCC cells [37]. Extracellular vesicles derived from tumor cells are critical mediators of cell-to-cell communication in the setting of tumorigenesis [66]. Ectosome PKM2 released by HCC cells facilitates monocyte-to-M2 macrophage differentiation via the STAT3 signaling pathway and remodels an immunosuppressive microenvironment, allowing immune escape and tumor progression [27]. Although PKM2 exhibits a dramatic promoting effect on HCC progression, the global ablation of PKM2 results in spontaneous tumor formation, highlighting the complexity of PKM2 in regulating HCC [67].

### 3.2. PKM2 in CCA

CCA is a highly lethal adenocarcinoma of the hepatobiliary system that is characterized by late diagnosis, short-term survival, and chemoresistance [68]. PKM2-associated aerobic glycolysis is also enhanced in CCA cells, resulting in low levels of pyruvate, a decreased inhibitory effect on HDAC3, and the suppression of apoptosis [69]. Furthermore, PKM2 is recognized as a key player in regulating EMT in CCA [70]. The knockdown of PKM2 effectively inhibits the migration, invasion, and proangiogenic capability of CCA cells via the downregulation of EMT-related markers [71]. Yu et al. provided in vivo evidence that PKM2 inhibition suppresses CCA cell proliferation, tumor growth, and neural invasion [38]. Moreover, the overexpression of CNRIP1 (a tumor suppressor) facilitated PKM2 degradation by activating parkin, which inhibited CCA progression in a mouse xenograft model [72]. These findings accentuate the potential of targeting PKM2 to combat CCA.

## 4. Inflammatory Liver Diseases

The liver is generally considered a vital organ that participates in metabolism, nutrient storage, and detoxification. During these complex processes, the hepatic immune system is challenged by numerous bacterial stimuli and harmful molecules. Maintaining homeostasis requires the liver to be immunotolerant while remaining alert to potential infectious agents or tissue damage [73]. Owing to these unique characteristics, the mechanisms that resolve hepatic inflammation are extremely important [74]. Failure to sustain tissue homeostasis leads to inflammation and liver injury, potentiating the development of fibrosis, cirrhosis, and even HCC.

### 4.1. PKM2 in Fatty Liver Diseases

**Figure 2:**
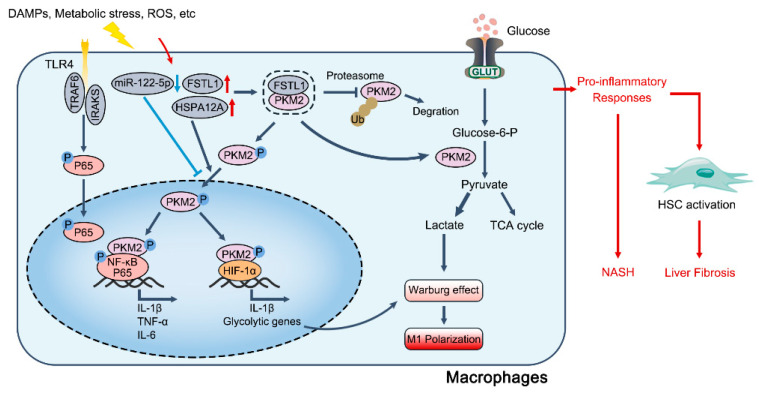
Role of macrophage PKM2 in the progression of NAFLD and liver fibrosis. During the pathogenesis of NASH and liver fibrosis, several stimuli released by injured hepatocytes activate hepatic macrophages via TLR4/NF-κB signaling. Meanwhile, levels of FSTL1 and HSPA12A are elevated when the expression of miR-122-5p is downregulated in response to liver inflammation, both of which are reported to regulate PKM2 Y105 phosphorylation and nuclear translocation. Specifically, FSTL1 directly binds to PKM2 and maintains its stability, thereby promoting PKM2-mediated glycolysis and dimer activity in M1 macrophages. On one hand, the nuclear translocation of PKM2 activates the NF-κB-directed and HIF-1α-directed transcription of pro-inflammatory genes including IL-1β. On the other hand, PKM2-HIF-1α transactivation upregulates the expression of several glycolytic genes, which further fuel aerobic glycolysis and macrophage M1 polarization. Ultimately, PKM2-mediated pro-inflammatory responses perpetuate hepatic inflammation and exacerbate the development of NASH and liver fibrosis.

Liver steatosis, which is attributed to obesity, alcohol use, or chemical-induced injury, may lead to fatty liver disease and further progress to steatohepatitis in the presence of inflammation [75]. During this process, M1 macrophages exacerbate hepatic inflammation and disease progression, whereas M2 macrophages protect against steatosis and liver fibrosis [76]. Particularly, the PKM2-driven progression of fatty liver disease is mainly dependent on metabolic reprogramming and the M1 polarization of hepatic macrophages (Figure 2). PKM2-mediated glycolysis is enhanced during macrophage M1 polarization in NASH, which correlates with miR-122-5p downregulation [40]. Kong found that HSPA12A binds to PKM2 and stimulates its nuclear translocation, which further provokes macrophage M1 polarization and the secretion of pro-inflammatory cytokines, including IL-1β and IL-6, ultimately leading to hepatocyte steatosis via paracrine effects [77]. PKM2 is also upregulated in hepatocytes during steatosis, and the disruption of PKM2 activity alleviates mitochondrial ROS and hepatocyte lipid accumulation [24]. Moreover, PKM2 has been shown to regulate the metabolic skewing of Th17 cells, and cell-specific PKM2 knockout effectively ameliorates hepatic inflammation and NAFLD [41]. In ALD, hepatocyte DRAM1 is upregulated in response to excessive ethanol, which increases PKM2-enriched extracellular vesicles, thereby promoting macrophage M1 activation and hepatic inflammation [78].

### 4.2. PKM2 in Liver Fibrosis and Cirrhosis

PKM2 is involved in the progression of liver fibrosis, which is a major cause of mortality in patients with end-stage liver disease, and it is characterized by hepatocyte injury and HSC (hepatic stellate cell) activation. Macrophages play an important role in perpetuating hepatic inflammation and HSC activation via the release of pro-inflammatory cytokines [79]. Rao et al. found that FSTL1 promotes PKM2 stability and nuclear translocation in macrophages, which further enhances macrophage M1 polarization, the production of pro-inflammatory cytokines, HSC activation, and liver fibrosis [80]. PKM2 in HSC also promotes its activation and fibrogenesis by facilitating aerobic glycolysis by regulating histone H3K9 acetylation in activated HSCs [42]. Interestingly, activated HSC can release PKM2-enriched exosomes that induce the glycolysis and activation of quiescent HSCs, hepatic macrophages, and LSECs, forming a positive feedback loop that promotes the progression of liver fibrosis [81].

## 5. Therapeutic Opportunities of PKM2-Targeted Therapy 

PKM2-targeting molecules have been mainly characterized as inhibitors and agonists (Table 1). When inhibitors limit PKM2 tetramer formation, agonists induce the transformation of PKM2 dimers into tetramers, thereby limiting its nuclear translocation [82,83]. Although both inhibitors and agonists can inhibit PKM2-mediated glycolysis and immune activation, whether an inhibitor could affect PKM2 nuclear translocation remains incompletely understood [58,59,84,85,86]. Nevertheless, treatments including traditional Chinese medicine or nucleotides-related therapeutics have been shown to modulate PKM2 activity in CLDs. In this section, we highlight the translational potential of PKM2-targeting therapy in combating CLDs. 

In neoplastic liver diseases, the inhibition of PKM2 by either shikonin or compound 3k suppresses glycolysis and proliferation, induces apoptosis in HCC cells in vitro and enhances the antitumor effect of sorafenib in vivo [87,88,98]. Similarly, shikonin has been reported to inhibit the growth and migration of CCA cells in vitro, whereas the in vivo evidence remains lacking [90,91]. Meanwhile, shikonin aggravates the oxidative stress and nutrient deficiency of HCC cells by causing mitochondria dysfunction, which further validates the efficacy of PKM2 inhibition in treating HCC [99]. Transarterial chemoembolization (TACE) is a palliative and neoadjuvant treatment for HCC patients [100]. The upregulation of PKM2 is strongly associated with a decreased response rate and shortened survival in patients receiving TACE, whereas the inhibition of PKM2 by shikonin effectively improves the efficacy of TACE in resistant cells [101]. Notably, Lu et al. reported that shikonin unexpectedly induced PKM2 nuclear translocation and the transcription of BAG3, a gene related to sustained cell survival, suggesting that a combination of a BAG3 inhibitor and shikonin may exhibit better anti-HCC efficacy [85]. The PKM2 activator can also be used to treat HCC. Unlike inhibitors, PKM2 activators display antitumor effects by enhancing pyruvate kinase activity, resulting in complete glycolysis and decreased anabolism, thereby inhibiting the growth of solid tumors including HCC [102,103]. In addition to the PKM2 inhibitor and activator, protein hydrolysate extracted from Oviductus Ranae reduces PKM2 expression by upregulating miR-491-5p and thereby efficiently prohibited HCC growth and metastasis [104]. Moreover, PKM2 shifts metabolites to aerobic glycolysis, whereas PKM1 drives metabolism toward oxidative phosphorylation. An antisense oligonucleotide (ASO) that switches *PKM* splicing from tumor-promoting PKM2 to the PKM1 isoform limits aerobic glycolysis, thereby inhibiting HCC growth both in vitro and in vivo, laying the groundwork for a potential ASO-based splicing therapy in treating liver cancer [105,106].

PKM2 is also a promising target for the treatment of inflammatory CLDs. Gwon et al. discovered that shikonin attenuates HFD-induced NAFLD by stimulating fatty acid oxidation and energy expenditure via AMPK activation [92]. Tong et al. found that shikonin can alleviate liver fibrosis by downregulating the TGF-β1/Smad pathway [93]. Although the role of PKM2 was not emphasized in the above studies, PKM2 is closely related to mitochondrial fitness and autophagy [107]. Therefore, the therapeutic efficacy of shikonin in NAFLD and liver fibrosis may be partially attributed to alterations in PKM2 activity. Furthermore, pharmacological PKM2 agonists, which limit PKM2 nuclear translocation, effectively ameliorate MCD-induced NASH in mice by re-educating macrophages from M1 to M2 polarization [95]. Annexin A5 attenuates HFD-induced NASH by regulating hepatic macrophage polarization by directly blocking PKM2 Y105 phosphorylation and nuclear translocation [108]. Digoxin, a cardiac glycoside, ameliorates steatohepatitis by disrupting PKM2–HIF-1α transactivation, thereby inhibiting metabolic reprogramming and the pro-inflammatory activation of macrophages [96]. A plant-derived triterpene celastrol that limits glycolysis and reprograms macrophage polarization from the pro-inflammatory M1 phenotype to the anti-inflammatory M2 phenotype was found to simultaneously restrain PKM2 nuclear translocation and enzymatic activity at the same time and protect against NAFLD [109]. A recent study also demonstrated that lapachol ameliorates NAFLD progression by directly inhibiting PKM2 phosphorylation and nuclear translocation, which then suppresses Kupffer cell M1 polarization [110]. Furthermore, PKM2 is involved in HSC activation, and limiting PKM2 nuclear translocation by TEPP-46 effectively attenuates the progression of liver fibrosis by inhibiting HSC activation [42,94]. These studies feature PKM2 as an attractive pharmacological target in treating CLDs. 

## 6. Conclusions and Future Perspectives

Chronic liver diseases (CLDs) encompass a broad spectrum of liver diseases ranging from ALD and NAFLD to life-threatening NASH, cirrhosis, and even HCC. The incidence of most CLDs is continuously rising when effective or approved treatments are lacking. The Warburg effect (aerobic glycolysis) plays an important role in the progression of CLDs [111,112]. In neoplastic CLDs, including HCC and CCA, the Warburg effect fuels and sustains tumor growth, metastasis, recurrence, and drug resistance [113]. In non-neoplastic CLDs, the Warburg effect is tightly linked to immune activation and hepatic inflammation, which is a condition that is profoundly involved in NASH and liver fibrosis [114,115,116]. Herein, understanding the mechanisms governing the Warburg effect in CLDs may help identify novel therapeutic targets.

PKM2 is a rate-limiting enzyme in glycolysis. Owing to its unique dimeric form with low pyruvate kinase activity, the upregulation of PKM2 is a hallmark of cells with increased aerobic glycolysis. Although numerous studies have attempted to elucidate the importance of PKM2 in the development of neoplastic diseases, its role of PKM2 in inflammatory disorders, especially CLDs, has not been fully elucidated. The translation of this knowledge into clinical practice is at a nascent stage, partly owing to the lack of studies assessing the cell-specific role of PKM2 in CLDs, as the function of PKM2 in certain hepatic cells, including LSECs and bile duct cells, remains elusive. Moreover, most studies that have investigated the role of PKM2 were based on cellular and animal models, which leaves the question of whether targeting PKM2 in human diseases will bring up beneficial effects similar to what has been observed in in vivo models. In silico models, including quantitative systems pharmacology (QSP) models, have been extensively applied to drug discovery by illustrating the molecular interactions between biological systems and drug candidates [117,118]. In particular, several well-established QSP models can be used to study glucose metabolism [119,120], the Warburg effect [121] and liver function [122], all of which may help further the translation study of PKM2 in combating CLDs.

Undoubtedly, as a therapeutic target, PKM2 has unique advantages, since its expression is almost undetectable in the healthy liver and starts to increase as the disease progresses. Furthermore, when cells undergo abnormal activation, PKM2 is mainly localized to the nucleus, potentiating the application of PKM2 activators in treating CLDs without affecting normal or quiescent cells. In conclusion, although future studies are required to illustrate the clinical significance of PKM2 targeting molecules along with their immediate and long-term health effects, PKM2 may serve as a novel therapeutic target for both neoplastic and inflammatory CLDs.

## Figures and Tables

**Table 1:** Main characteristics of PKM2-targeting compounds as CLD therapy.

Name	Structure	MW	Disease Types	Pharmacological Properties	Refs.
C3k	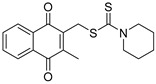	345.5	HCC	Inhibitor	[87,88]
Shikonin	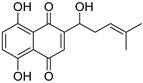	288.3	HCC	Inhibitor	[59,89]
			CCA		[90,91]
			NAFLD		[92]
			LF		[93]
ML265	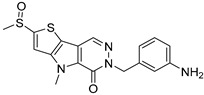	372.5	LF	Agonist	[42,94]
			NASH		[95]
DASA-58	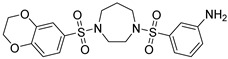	453.5	LF	Agonist	[80]
Digoxin	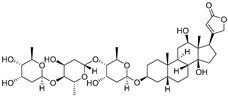	780.9	NASH	Inhibiting PKM2 trans-activation	[24,96]
Meformin	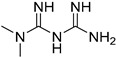	315.8	HCC	Suppressing PKM2 activity	[97]
			CCA		[38]

## Data Availability

Not applicable.

## Abbreviations

2-DG	2-deoxy-D-glucose
ALD	alcoholic liver disease
AMPK	AMP-activated protein kinase
BAG3	BAG cochaperone 3
CLD	chronic liver disease
CCA	cholangiocarcinoma
CCL20 C-C	motif chemokine ligand 20
CNRIP1	cannabinoid receptor interacting protein 1
DRAM1	DNA damage regulated autophagy modulator 1
EMT	Epithelial–mesenchymal transition
FSTL1	follistatin-like 1
GLUT1	glucose transporter protein type 1
GTPBP4	GTP binding protein 4
HCC	hepatocellular carcinoma
HSC	hepatic stellate cell
HSP90	heat shock protein 90
HIF-1α	hypoxia-inducible factor 1 alpha
HSPA12A	heat shock protein family A member 12A
HFD	high-fat diet
ICI	immune-checkpoint inhibitor
LSEC	liver sinusoidal endothelial cell
LF	liver fibrosis
MAT2B	methionine adenosyltransferase II beta
MyD88	myeloid differentiation primary response 88
MCD	methionine-choline deficient diet
NAFLD	nonalcoholic fatty liver disease
NASH	nonalcoholic steatohepatitis
OXPHOS	oxidative phosphorylation
PD-L1	programmed death-ligand 1
PRMT6	protein arginine N-methyltransferase 6
STAT3	signal transducer and activator of transcription 3
SMADS	suppressor of mothers against decapentaplegic
Th17	T helper 17
TACE	trans-arterial chemoembolization
TGF-β1	transforming growth factor beta 1

## References

[1] Moon A.M., Singal A.G., Tapper E.B. (2020). Contemporary Epidemiology of Chronic Liver Disease and Cirrhosis. Clin. Gastroenterol. Hepatol..

[2] Powell E.E., Wong V.W., Rinella M. (2021). Non-alcoholic fatty liver disease. Lancet.

[3] Ratziu V., Francque S., Sanyal A. (2022). Breakthroughs in therapies for NASH and remaining challenges. J. Hepatol..

[4] Friedman S.L., Pinzani M. (2022). Hepatic fibrosis 2022: Unmet needs and a blueprint for the future. Hepatology.

[5] Han H.S., Kang G., Kim J.S., Choi B.H., Koo S.H. (2016). Regulation of glucose metabolism from a liver-centric perspective. Exp. Mol. Med..

[6] Petersen M.C., Vatner D.F., Shulman G.I. (2017). Regulation of hepatic glucose metabolism in health and disease. Nat. Rev. Endocrinol..

[7] Feng J., Li J., Wu L., Yu Q., Ji J., Wu J., Dai W., Guo C. (2020). Emerging roles and the regulation of aerobic glycolysis in hepatocellular carcinoma. J. Exp. Clin. Cancer Res..

[8] Kubes P., Mehal W.Z. (2012). Sterile inflammation in the liver. Gastroenterology.

[9] El Kasmi K.C., Stenmark K.R. (2015). Contribution of metabolic reprogramming to macrophage plasticity and function. Semin. Immunol..

[10] Shang R.Z., Qu S.B., Wang D.S. (2016). Reprogramming of glucose metabolism in hepatocellular carcinoma: Progress and prospects. World J. Gastroenterol..

[11] Rui L., Lin J.D. (2022). Reprogramming of Hepatic Metabolism and Microenvironment in Nonalcoholic Steatohepatitis. Annu. Rev. Nutr..

[12] Jin T., Wang C., Tian Y., Dai C., Zhu Y., Xu F. (2020). Mitochondrial metabolic reprogramming: An important player in liver cancer progression. Cancer Lett..

[13] Delgado M.E., Cárdenas B.I., Farran N., Fernandez M. (2021). Metabolic Reprogramming of Liver Fibrosis. Cells.

[14] Raggi C., Taddei M.L., Rae C., Braconi C., Marra F. (2022). Metabolic reprogramming in cholangiocarcinoma. J. Hepatol..

[15] Alquraishi M., Puckett D.L., Alani D.S., Humidat A.S., Frankel V.D., Donohoe D.R., Whelan J., Bettaieb A. (2019). Pyruvate kinase M2: A simple molecule with complex functions. Free. Radic. Biol. Med..

[16] Zhang Z., Deng X., Liu Y., Liu Y., Sun L., Chen F. (2019). PKM2, function and expression and regulation. Cell. Biosci..

[17] Wong N., Ojo D., Yan J., Tang D. (2015). PKM2 contributes to cancer metabolism. Cancer Lett..

[18] Yang W., Zheng Y., Xia Y., Ji H., Chen X., Guo F., Lyssiotis C.A., Aldape K., Cantley L.C., Lu Z. (2012). ERK1/2-dependent phosphorylation and nuclear translocation of PKM2 promotes the Warburg effect. Nat. Cell. Biol..

[19] Palsson-McDermott E.M., Curtis A.M., Goel G., Lauterbach M.A., Sheedy F.J., Gleeson L.E., van den Bosch M.W., Quinn S.R., Domingo-Fernandez R., Johnston D.G. (2015). Pyruvate kinase M2 regulates Hif-1α activity and IL-1β induction and is a critical determinant of the warburg effect in LPS-activated macrophages. Cell Metab..

[20] Dayton T.L., Jacks T., Vander Heiden M.G. (2016). PKM2, cancer metabolism, and the road ahead. EMBO Rep..

[21] Zhu S., Guo Y., Zhang X., Liu H., Yin M., Chen X., Peng C. (2021). Pyruvate kinase M2 (PKM2) in cancer and cancer therapeutics. Cancer Lett..

[22] Liu C., Liu C., Fu R. (2022). Research progress on the role of PKM2 in the immune response. Front. Immunol..

[23] Patel S., Das A., Meshram P., Sharma A., Chowdhury A., Jariyal H., Datta A., Sarmah D., Nalla L.V., Sahu B. (2021). Pyruvate kinase M2 in chronic inflammations: A potpourri of crucial protein-protein interactions. Cell. Biol. Toxicol..

[24] Zhao P., Han S.N., Arumugam S., Yousaf M.N., Qin Y., Jiang J.X., Torok N.J., Chen Y., Mankash M.S., Liu J. (2019). Digoxin improves steatohepatitis with differential involvement of liver cell subsets in mice through inhibition of PKM2 transactivation. Am. J. Physiol. Gastrointest. Liver Physiol..

[25] Liu J., Zhang Q., Chen H., Gao Z., Li Y., Sun Z., Xiang R., Zhang S. (2016). Phage display library selection of a hypoxia-binding scFv antibody for liver cancer metabolic marker discovery. Oncotarget.

[26] Bluemlein K., Grüning N.M., Feichtinger R.G., Lehrach H., Kofler B., Ralser M. (2011). No evidence for a shift in pyruvate kinase PKM1 to PKM2 expression during tumorigenesis. Oncotarget.

[27] Hou P.P., Luo L.J., Chen H.Z., Chen Q.T., Bian X.L., Wu S.F., Zhou J.X., Zhao W.X., Liu J.M., Wang X.M. (2020). Ectosomal PKM2 Promotes HCC by Inducing Macrophage Differentiation and Remodeling the Tumor Microenvironment. Mol. Cell.

[28] Li T.E., Wang S., Shen X.T., Zhang Z., Chen M., Wang H., Zhu Y., Xu D., Hu B.Y., Wei R. (2020). PKM2 Drives Hepatocellular Carcinoma Progression by Inducing Immunosuppressive Microenvironment. Front. Immunol..

[29] Lv W.W., Liu D., Liu X.C., Feng T.N., Li L., Qian B.Y., Li W.X. (2018). Effects of PKM2 on global metabolic changes and prognosis in hepatocellular carcinoma: From gene expression to drug discovery. BMC Cancer.

[30] Zhao R., Li L., Yang J., Niu Q., Wang H., Qin X., Zhu N., Shi A. (2020). Overexpression of Pyruvate Kinase M2 in Tumor Tissues Is Associated with Poor Prognosis in Patients with Hepatocellular Carcinoma. Pathol. Oncol. Res..

[31] Chen Z., Lu X., Wang Z., Jin G., Wang Q., Chen D., Chen T., Li J., Fan J., Cong W. (2015). Co-expression of PKM2 and TRIM35 predicts survival and recurrence in hepatocellular carcinoma. Oncotarget.

[32] Tai W.T., Hung M.H., Chu P.Y., Chen Y.L., Chen L.J., Tsai M.H., Chen M.H., Shiau C.W., Boo Y.P., Chen K.F. (2016). SH2 domain-containing phosphatase 1 regulates pyruvate kinase M2 in hepatocellular carcinoma. Oncotarget.

[33] Harris P.S., Hansen R.M., Gray M.E., Massoud O.I., McGuire B.M., Shoreibah M.G. (2019). Hepatocellular carcinoma surveillance: An evidence-based approach. World J. Gastroenterol..

[34] Lee N.C.W., Carella M.A., Papa S., Bubici C. (2018). High Expression of Glycolytic Genes in Cirrhosis Correlates With the Risk of Developing Liver Cancer. Front. Cell. Dev. Biol..

[35] Liu Y., Wu H., Mei Y., Ding X., Yang X., Li C., Deng M., Gong J. (2017). Clinicopathological and prognostic significance of PKM2 protein expression in cirrhotic hepatocellular carcinoma and non-cirrhotic hepatocellular carcinoma. Sci. Rep..

[36] Zhu Y., Yang J., Xu D., Gao X.M., Zhang Z., Hsu J.L., Li C.W., Lim S.O., Sheng Y.Y., Zhang Y. (2019). Disruption of tumour-associated macrophage trafficking by the osteopontin-induced colony-stimulating factor-1 signalling sensitises hepatocellular carcinoma to anti-PD-L1 blockade. Gut.

[37] Lu L.G., Zhou Z.L., Wang X.Y., Liu B.Y., Lu J.Y., Liu S., Zhang G.B., Zhan M.X., Chen Y. (2022). PD-L1 blockade liberates intrinsic antitumourigenic properties of glycolytic macrophages in hepatocellular carcinoma. Gut.

[38] Yu G., Yu W., Jin G., Xu D., Chen Y., Xia T., Yu A., Fang W., Zhang X., Li Z. (2015). PKM2 regulates neural invasion of and predicts poor prognosis for human hilar cholangiocarcinoma. Mol. Cancer.

[39] Cuenco J., Wehnert N., Blyuss O., Kazarian A., Whitwell H.J., Menon U., Dawnay A., Manns M.P., Pereira S.P., Timms J.F. (2018). Identification of a serum biomarker panel for the differential diagnosis of cholangiocarcinoma and primary sclerosing cholangitis. Oncotarget.

[40] Inomata Y., Oh J.W., Taniguchi K., Sugito N., Kawaguchi N., Hirokawa F., Lee S.W., Akao Y., Takai S., Kim K.P. (2022). Downregulation of miR-122-5p Activates Glycolysis via PKM2 in Kupffer Cells of Rat and Mouse Models of Non-Alcoholic Steatohepatitis. Int. J. Mol. Sci..

[41] Moreno-Fernandez M.E., Giles D.A., Oates J.R., Chan C.C., Damen M., Doll J.R., Stankiewicz T.E., Chen X., Chetal K., Karns R. (2021). PKM2-dependent metabolic skewing of hepatic Th17 cells regulates pathogenesis of non-alcoholic fatty liver disease. Cell. Metab..

[42] Zheng D., Jiang Y., Qu C., Yuan H., Hu K., He L., Chen P., Li J., Tu M., Lin L. (2020). Pyruvate Kinase M2 Tetramerization Protects against Hepatic Stellate Cell Activation and Liver Fibrosis. Am. J. Pathol..

[43] Meoli L., Gupta N.K., Saeidi N., Panciotti C.A., Biddinger S.B., Corey K.E., Stylopoulos N. (2018). Nonalcoholic fatty liver disease and gastric bypass surgery regulate serum and hepatic levels of pyruvate kinase isoenzyme M2. Am. J. Physiol. Endocrinol. Metab..

[44] Sheka A.C., Adeyi O., Thompson J., Hameed B., Crawford P.A., Ikramuddin S. (2020). Nonalcoholic Steatohepatitis: A Review. JAMA.

[45] Lurie Y., Webb M., Cytter-Kuint R., Shteingart S., Lederkremer G.Z. (2015). Non-invasive diagnosis of liver fibrosis and cirrhosis. World J. Gastroenterol..

[46] Li X., Ramadori P., Pfister D., Seehawer M., Zender L., Heikenwalder M. (2021). The immunological and metabolic landscape in primary and metastatic liver cancer. Nat. Rev. Cancer.

[47] Donne R., Lujambio A. (2023). The liver cancer immune microenvironment: Therapeutic implications for hepatocellular carcinoma. Hepatology.

[48] Huang D.Q., El-Serag H.B., Loomba R. (2021). Global epidemiology of NAFLD-related HCC: Trends, predictions, risk factors and prevention. Nat. Rev. Gastroenterol. Hepatol..

[49] Foerster F., Gairing S.J., Ilyas S.I., Galle P.R. (2022). Emerging immunotherapy for HCC: A guide for hepatologists. Hepatology.

[50] Pfister D., Núñez N.G., Pinyol R., Govaere O., Pinter M., Szydlowska M., Gupta R., Qiu M., Deczkowska A., Weiner A. (2021). NASH limits anti-tumour surveillance in immunotherapy-treated HCC. Nature.

[51] Xu Q., Tu J., Dou C., Zhang J., Yang L., Liu X., Lei K., Liu Z., Wang Y., Li L. (2017). HSP90 promotes cell glycolysis, proliferation and inhibits apoptosis by regulating PKM2 abundance via Thr-328 phosphorylation in hepatocellular carcinoma. Mol. Cancer.

[52] Li Q., Pan X., Zhu D., Deng Z., Jiang R., Wang X. (2019). Circular RNA MAT2B Promotes Glycolysis and Malignancy of Hepatocellular Carcinoma Through the miR-338-3p/PKM2 Axis Under Hypoxic Stress. Hepatology.

[53] Yu L., Kim J., Jiang L., Feng B., Ying Y., Ji K.Y., Tang Q., Chen W., Mai T., Dou W. (2020). MTR4 drives liver tumorigenesis by promoting cancer metabolic switch through alternative splicing. Nat. Commun..

[54] Liu B., Qu X., Wang J., Xu L., Zhang L., Xu B., Su J., Bian X. (2023). LINC00365 functions as a tumor suppressor by inhibiting HIF-1α-mediated glucose metabolism reprogramming in breast cancer. Exp. Cell. Res..

[55] Wang J.Z., Zhu W., Han J., Yang X., Zhou R., Lu H.C., Yu H., Yuan W.B., Li P.C., Tao J. (2021). The role of the HIF-1α/ALYREF/PKM2 axis in glycolysis and tumorigenesis of bladder cancer. Cancer Commun..

[56] Zhang X., Li Y., Ma Y., Yang L., Wang T., Meng X., Zong Z., Sun X., Hua X., Li H. (2018). Yes-associated protein (YAP) binds to HIF-1α and sustains HIF-1α protein stability to promote hepatocellular carcinoma cell glycolysis under hypoxic stress. J. Exp. Clin. Cancer Res..

[57] Luo W., Hu H., Chang R., Zhong J., Knabel M., O’Meally R., Cole R.N., Pandey A., Semenza G.L. (2011). Pyruvate kinase M2 is a PHD3-stimulated coactivator for hypoxia-inducible factor 1. Cell.

[58] Pan R.Y., He L., Zhang J., Liu X., Liao Y., Gao J., Liao Y., Yan Y., Li Q., Zhou X. (2022). Positive feedback regulation of microglial glucose metabolism by histone H4 lysine 12 lactylation in Alzheimer’s disease. Cell. Metab..

[59] Yang W., Liu J., Hou L., Chen Q., Liu Y. (2021). Shikonin differentially regulates glucose metabolism via PKM2 and HIF1α to overcome apoptosis in a refractory HCC cell line. Life Sci..

[60] Feng J., Dai W., Mao Y., Wu L., Li J., Chen K., Yu Q., Kong R., Li S., Zhang J. (2020). Simvastatin re-sensitizes hepatocellular carcinoma cells to sorafenib by inhibiting HIF-1α/PPAR-γ/PKM2-mediated glycolysis. J. Exp. Clin. Cancer Res..

[61] Wong T.L., Ng K.Y., Tan K.V., Chan L.H., Zhou L., Che N., Hoo R.L.C., Lee T.K., Richard S., Lo C.M. (2020). CRAF Methylation by PRMT6 Regulates Aerobic Glycolysis-Driven Hepatocarcinogenesis via ERK-Dependent PKM2 Nuclear Relocalization and Activation. Hepatology.

[62] Zhou Q., Yin Y., Yu M., Gao D., Sun J., Yang Z., Weng J., Chen W., Atyah M., Shen Y. (2022). GTPBP4 promotes hepatocellular carcinoma progression and metastasis via the PKM2 dependent glucose metabolism. Redox Biol..

[63] Yuan Q., Zhang J., Liu Y., Chen H., Liu H., Wang J., Niu M., Hou L., Wu Z., Chen Z. (2022). MyD88 in myofibroblasts regulates aerobic glycolysis-driven hepatocarcinogenesis via ERK-dependent PKM2 nuclear relocalization and activation. J. Pathol..

[64] Yu Z., Wang D., Tang Y. (2021). PKM2 promotes cell metastasis and inhibits autophagy via the JAK/STAT3 pathway in hepatocellular carcinoma. Mol. Cell. Biochem..

[65] Liu W.R., Tian M.X., Yang L.X., Lin Y.L., Jin L., Ding Z.B., Shen Y.H., Peng Y.F., Gao D.M., Zhou J. (2015). PKM2 promotes metastasis by recruiting myeloid-derived suppressor cells and indicates poor prognosis for hepatocellular carcinoma. Oncotarget.

[66] Li X., Li C., Zhang L., Wu M., Cao K., Jiang F., Chen D., Li N., Li W. (2020). The significance of exosomes in the development and treatment of hepatocellular carcinoma. Mol. Cancer.

[67] Dayton T.L., Gocheva V., Miller K.M., Israelsen W.J., Bhutkar A., Clish C.B., Davidson S.M., Luengo A., Bronson R.T., Jacks T. (2016). Germline loss of PKM2 promotes metabolic distress and hepatocellular carcinoma. Genes. Dev..

[68] Brindley P.J., Bachini M., Ilyas S.I., Khan S.A., Loukas A., Sirica A.E., Teh B.T., Wongkham S., Gores G.J. (2021). Cholangiocarcinoma. Nat. Rev. Dis. Primers.

[69] Zhang M., Pan Y., Tang D., Dorfman R.G., Xu L., Zhou Q., Zhou L., Wang Y., Li Y., Yin Y. (2019). Low levels of pyruvate induced by a positive feedback loop protects cholangiocarcinoma cells from apoptosis. Cell. Commun. Signal..

[70] Lei G.L., Li Z., Li Y.Y., Hong Z.X., Wang S., Bai Z.F., Sun F., Yan J., Yu L.X., Yang P.H. (2021). Long noncoding RNA FAM66C promotes tumor progression and glycolysis in intrahepatic cholangiocarcinoma by regulating hsa-miR-23b-3p/KCND2 axis. Environ. Toxicol..

[71] Peng C., Sun Z., Li O., Guo C., Yi W., Tan Z., Jiang B. (2019). Leptin stimulates the epithelial-mesenchymal transition and pro-angiogenic capability of cholangiocarcinoma cells through the miR-122/PKM2 axis. Int. J. Oncol..

[72] Chen D., Wu H., Feng X., Chen Y., Lv Z., Kota V.G., Chen J., Wu W., Lu Y., Liu H. (2021). DNA Methylation of Cannabinoid Receptor Interacting Protein 1 Promotes Pathogenesis of Intrahepatic Cholangiocarcinoma Through Suppressing Parkin-Dependent Pyruvate Kinase M2 Ubiquitination. Hepatology.

[73] Heymann F., Tacke F. (2016). Immunology in the liver--from homeostasis to disease. Nat. Rev. Gastroenterol. Hepatol..

[74] Robinson M.W., Harmon C., O’Farrelly C. (2016). Liver immunology and its role in inflammation and homeostasis. Cell. Mol. Immunol..

[75] Paul B., Lewinska M., Andersen J.B. (2022). Lipid alterations in chronic liver disease and liver cancer. JHEP Rep..

[76] Wang Q., Zhou H., Bu Q., Wei S., Li L., Zhou J., Zhou S., Su W., Liu M., Liu Z. (2022). Role of XBP1 in regulating the progression of non-alcoholic steatohepatitis. J. Hepatol..

[77] Kong Q., Li N., Cheng H., Zhang X., Cao X., Qi T., Dai L., Zhang Z., Chen X., Li C. (2019). HSPA12A Is a Novel Player in Nonalcoholic Steatohepatitis via Promoting Nuclear PKM2-Mediated M1 Macrophage Polarization. Diabetes.

[78] Tan J., Zhang J., Wang M., Wang Y., Dong M., Ma X., Sun B., Liu S., Zhao Z., Chen L. (2022). DRAM1 increases the secretion of PKM2-enriched EVs from hepatocytes to promote macrophage activation and disease progression in ALD. Mol. Ther. Nucleic Acids.

[79] Kisseleva T., Brenner D. (2021). Molecular and cellular mechanisms of liver fibrosis and its regression. Nat. Rev. Gastroenterol. Hepatol..

[80] Rao J., Wang H., Ni M., Wang Z., Wang Z., Wei S., Liu M., Wang P., Qiu J., Zhang L. (2022). FSTL1 promotes liver fibrosis by reprogramming macrophage function through modulating the intracellular function of PKM2. Gut.

[81] Wan L., Xia T., Du Y., Liu J., Xie Y., Zhang Y., Guan F., Wu J., Wang X., Shi C. (2019). Exosomes from activated hepatic stellate cells contain GLUT1 and PKM2: A role for exosomes in metabolic switch of liver nonparenchymal cells. FASEB J..

[82] Chen J., Xie J., Jiang Z., Wang B., Wang Y., Hu X. (2011). Shikonin and its analogs inhibit cancer cell glycolysis by targeting tumor pyruvate kinase-M2. Oncogene.

[83] Anastasiou D., Yu Y., Israelsen W.J., Jiang J.K., Boxer M.B., Hong B.S., Tempel W., Dimov S., Shen M., Jha A. (2012). Pyruvate kinase M2 activators promote tetramer formation and suppress tumorigenesis. Nat. Chem. Biol..

[84] Huang B., Wang Q., Jiang L., Lu S., Li C., Xu C., Wang C., Zhang E., Zhang X. (2022). Shikonin ameliorated mice colitis by inhibiting dimerization and tetramerization of PKM2 in macrophages. Front. Pharmacol..

[85] Lu J., Liu S.Y., Zhang J., Yang G.M., Gao G.B., Yu N.N., Li Y.P., Li Y.X., Ma Z.Q., Wang Y. (2021). Inhibition of BAG3 enhances the anticancer effect of shikonin in hepatocellular carcinoma. Am. J. Cancer Res..

[86] Angiari S., Runtsch M.C., Sutton C.E., Palsson-McDermott E.M., Kelly B., Rana N., Kane H., Papadopoulou G., Pearce E.L., Mills K.H.G. (2020). Pharmacological Activation of Pyruvate Kinase M2 Inhibits CD4(+) T Cell Pathogenicity and Suppresses Autoimmunity. Cell. Metab..

[87] Pang Y., Lin Y., Wang X., Wang J., Liu Q., Ding N., Huang L., Xiang Q., Fang J., Tan G. (2022). Inhibition of abnormally activated HIF-1α-GLUT1/3-glycolysis pathway enhances the sensitivity of hepatocellular carcinoma to 5-caffeoylquinic acid and its derivatives. Eur. J. Pharmacol..

[88] Zeng Z., Lan J., Lei S., Yang Y., He Z., Xue Y., Chen T. (2020). Simultaneous Inhibition of Ornithine Decarboxylase 1 and Pyruvate Kinase M2 Exerts Synergistic Effects Against Hepatocellular Carcinoma Cells. Onco Targets Ther..

[89] Zhang J., Shang L., Jiang W., Wu W. (2022). Shikonin induces apoptosis and autophagy via downregulation of pyrroline-5-carboxylate reductase1 in hepatocellular carcinoma cells. Bioengineered.

[90] Thonsri U., Seubwai W., Waraasawapati S., Wongkham S., Boonmars T., Cha’on U., Wongkham C. (2020). Antitumor Effect of Shikonin, a PKM2 Inhibitor, in Cholangiocarcinoma Cell Lines. Anticancer Res..

[91] Zhou G., Yang Z., Wang X., Tao R., Zhou Y. (2017). TRAIL Enhances Shikonin Induced Apoptosis through ROS/JNK Signaling in Cholangiocarcinoma Cells. Cell. Physiol. Biochem..

[92] Gwon S.Y., Ahn J., Jung C.H., Moon B., Ha T.Y. (2020). Shikonin Attenuates Hepatic Steatosis by Enhancing Beta Oxidation and Energy Expenditure via AMPK Activation. Nutrients.

[93] Liu T., Xu L., Wang C., Chen K., Xia Y., Li J., Li S., Wu L., Feng J., Xu S. (2019). Alleviation of hepatic fibrosis and autophagy via inhibition of transforming growth factor-β1/Smads pathway through shikonin. J. Gastroenterol. Hepatol..

[94] Satyanarayana G., Turaga R.C., Sharma M., Wang S., Mishra F., Peng G., Deng X., Yang J., Liu Z.R. (2021). Pyruvate kinase M2 regulates fibrosis development and progression by controlling glycine auxotrophy in myofibroblasts. Theranostics.

[95] Yang S.H., Wu H., Yi Z.J., Lai X. (2021). The PKM2 activator TEPP-46 attenuates MCD feeding-induced nonalcoholic steatohepatitis by inhibiting the activation of Kupffer cells. Eur. Rev. Med. Pharmacol. Sci..

[96] Ouyang X., Han S.N., Zhang J.Y., Dioletis E., Nemeth B.T., Pacher P., Feng D., Bataller R., Cabezas J., Stärkel P. (2018). Digoxin Suppresses Pyruvate Kinase M2-Promoted HIF-1α Transactivation in Steatohepatitis. Cell. Metab..

[97] Zhang C., Hu J., Sheng L., Yuan M., Wu Y., Chen L., Zheng G., Qiu Z. (2019). Metformin delays AKT/c-Met-driven hepatocarcinogenesis by regulating signaling pathways for de novo lipogenesis and ATP generation. Toxicol. Appl. Pharmacol..

[98] Liu T., Li S., Wu L., Yu Q., Li J., Feng J., Zhang J., Chen J., Zhou Y., Ji J. (2020). Experimental Study of Hepatocellular Carcinoma Treatment by Shikonin Through Regulating PKM2. J. Hepatocell. Carcinoma.

[99] Liu B., Jin J., Zhang Z., Zuo L., Jiang M., Xie C. (2019). Shikonin exerts antitumor activity by causing mitochondrial dysfunction in hepatocellular carcinoma through PKM2-AMPK-PGC1α signaling pathway. Biochem. Cell Biol..

[100] Sieghart W., Hucke F., Peck-Radosavljevic M. (2015). Transarterial chemoembolization: Modalities, indication, and patient selection. J. Hepatol..

[101] Martin S.P., Fako V., Dang H., Dominguez D.A., Khatib S., Ma L., Wang H., Zheng W., Wang X.W. (2020). PKM2 inhibition may reverse therapeutic resistance to transarterial chemoembolization in hepatocellular carcinoma. J. Exp. Clin. Cancer Res..

[102] Li Z., Zheng W., Li H., Li C., Gong Z. (2015). Synergistic Induction of Potential Warburg Effect in Zebrafish Hepatocellular Carcinoma by Co-Transgenic Expression of Myc and xmrk Oncogenes. PLoS ONE.

[103] Pathi S., Peterson P., Mangelson R., Tyagi E., Foulks J.M., Whatcott C.J., Bearss D.J., Warner S.L. (2019). Abstract B080: PKM2 activation modulates metabolism and enhances immune response in solid tumor models. Mol. Cancer Ther..

[104] Xu Q., Dou C., Liu X., Yang L., Ni C., Wang J., Guo Y., Yang W., Tong X., Huang D. (2018). Oviductus ranae protein hydrolysate (ORPH) inhibits the growth, metastasis and glycolysis of HCC by targeting miR-491-5p/PKM2 axis. Biomed. Pharmacother..

[105] Ma W.K., Voss D.M., Scharner J., Costa A.S.H., Lin K.T., Jeon H.Y., Wilkinson J.E., Jackson M., Rigo F., Bennett C.F. (2022). ASO-Based PKM Splice-Switching Therapy Inhibits Hepatocellular Carcinoma Growth. Cancer Res..

[106] Wang Z., Jeon H.Y., Rigo F., Bennett C.F., Krainer A.R. (2012). Manipulation of PK-M mutually exclusive alternative splicing by antisense oligonucleotides. Open. Biol..

[107] Liang J., Cao R., Wang X., Zhang Y., Wang P., Gao H., Li C., Yang F., Zeng R., Wei P. (2017). Mitochondrial PKM2 regulates oxidative stress-induced apoptosis by stabilizing Bcl2. Cell Res..

[108] Xu F., Guo M., Huang W., Feng L., Zhu J., Luo K., Gao J., Zheng B., Kong L.D., Pang T. (2020). Annexin A5 regulates hepatic macrophage polarization via directly targeting PKM2 and ameliorates NASH. Redox Biol..

[109] Fan N., Zhang X., Zhao W., Zhao J., Luo D., Sun Y., Li D., Zhao C., Wang Y., Zhang H. (2022). Covalent Inhibition of Pyruvate Kinase M2 Reprograms Metabolic and Inflammatory Pathways in Hepatic Macrophages against Non-alcoholic Fatty Liver Disease. Int. J. Biol. Sci..

[110] Yang Y., Sheng J., Sheng Y., Wang J., Zhou X., Li W., Kong Y. (2023). Lapachol treats non-alcoholic fatty liver disease by modulating the M1 polarization of Kupffer cells via PKM2. Int. Immunopharmacol..

[111] Zhou Y., Lin F., Wan T., Chen A., Wang H., Jiang B., Zhao W., Liao S., Wang S., Li G. (2021). ZEB1 enhances Warburg effect to facilitate tumorigenesis and metastasis of HCC by transcriptionally activating PFKM. Theranostics.

[112] Tarasenko T.N., Jestin M., Matsumoto S., Saito K., Hwang S., Gavrilova O., Trivedi N., Zerfas P.M., Barca E., DiMauro S. (2019). Macrophage derived TNFα promotes hepatic reprogramming to Warburg-like metabolism. J. Mol. Med..

[113] Beyoğlu D., Idle J.R. (2013). The metabolomic window into hepatobiliary disease. J. Hepatol..

[114] Dolin C.E., Arteel G.E. (2020). The Matrisome, Inflammation, and Liver Disease. Semin. Liver Dis..

[115] Seen S. (2021). Chronic liver disease and oxidative stress—A narrative review. Expert. Rev. Gastroenterol. Hepatol..

[116] Kelly B., O’Neill L.A. (2015). Metabolic reprogramming in macrophages and dendritic cells in innate immunity. Cell Res..

[117] Sové R.J., Verma B.K., Wang H., Ho W.J., Yarchoan M., Popel A.S. (2022). Virtual clinical trials of anti-PD-1 and anti-CTLA-4 immunotherapy in advanced hepatocellular carcinoma using a quantitative systems pharmacology model. J. Immunother. Cancer.

[118] Nijsen M., Wu F., Bansal L., Bradshaw-Pierce E., Chan J.R., Liederer B.M., Mettetal J.T., Schroeder P., Schuck E., Tsai A. (2018). Preclinical QSP Modeling in the Pharmaceutical Industry: An IQ Consortium Survey Examining the Current Landscape. CPT Pharmacomet. Syst. Pharmacol..

[119] Jeon M., Kang H.W., An S. (2018). A Mathematical Model for Enzyme Clustering in Glucose Metabolism. Sci. Rep..

[120] Mulukutla B.C., Yongky A., Daoutidis P., Hu W.S. (2014). Bistability in glycolysis pathway as a physiological switch in energy metabolism. PLoS ONE.

[121] Kapuy O., Makk-Merczel K., Szarka A. (2021). Therapeutic Approach of KRAS Mutant Tumours by the Combination of Pharmacologic Ascorbate and Chloroquine. Biomolecules.

[122] Verma B.K., Subramaniam P., Vadigepalli R. (2019). Model-based virtual patient analysis of human liver regeneration predicts critical perioperative factors controlling the dynamic mode of response to resection. BMC Syst. Biol..

